# Systematic Screening of Reference Genes for qPCR in *Procambarus clarkii*: *RPL13a* Shows Stable Expression Under Non-Ionic Ammonia Stress and Multiple Experimental Conditions

**DOI:** 10.3390/biology15201835

**Published:** 2026-10-13

**Authors:** Xiudan Yuan, Xing Tian, Yuanyuan Liu, Zhigang He, Jinlong Li, Yunpeng Fan, Jiarong Guo, Yong He, Shiming Deng, Dongwu Wang

**Affiliations:** 1 Hunan Fisheries Research Institute and Aquatic Products Seed Stock Station, Changsha 410153, China; xiudanyuan@hunaas.cn (X.Y.); tianx2323@163.com (X.T.); oceanhzg@163.com (Z.H.); wsljl2007@163.com (J.L.); guojr9913@163.com (J.G.); heyong19891989@outlook.com (Y.H.); 2 Yuelushan Laboratory, Changsha 410128, China; 3 College of Life Sciences, Hunan Normal University, Changsha 410081, China; 19174109653@163.com; 4 Agriculture Ministry Key Laboratory of Healthy Freshwater Aquaculture, Key Laboratory of Fish Health and Nutrition of Zhejiang Province, Zhejiang Institute of Freshwater Fisheries, Huzhou 313001, China; yunpengfan1994@163.com

**Keywords:** *Procambarus clarkii*, non-ionic ammonia, reference gene, real-time fluorescence quantitative PCR, *RPL13a*

## Abstract

Real-time quantitative PCR (qPCR) is a key technique for elucidating the mechanisms underlying environmental stress responses in aquatic animals, and its accuracy relies heavily on stable reference genes for data normalization. In this study, histopathological examination revealed alterations in the gill, hepatopancreas, and muscle tissues of *Procambarus clarkii* following NH_3_ stress. Based on transcriptomic screening and reference genes reported in the literature, 13 candidate reference genes were selected. Their expression levels in 8 different tissues before and after NH_3_ stress were detected using qPCR, and their stability was systematically evaluated using multiple algorithms, including geNorm, NormFinder, BestKeeper, Delta CT and RefFinder. The results identified 60S ribosomal protein L13a (*RPL13a*), ribosomal protein S18 (*RPS18*) and eukaryotic translation initiation factor 4A (*EIF4A*) as the three most stable candidates under NH_3_ stress, whereas the traditionally used reference genes beta-actin (*β-actin*) and glyceraldehyde 3-phosphate dehydrogenase (*GAPDH*) showed relatively low stability under these conditions. geNorm pairwise variation analysis indicated that the inclusion of a third reference gene could further improve normalization. Validation using four NH_3_-responsive genes showed that (*RPL13a* + *RPS18*) and (*RPL13a* + *RPS18* + *EIF4A*) yielded similar log_2_ fold-change estimates, supporting their use as normalization options under the tested conditions. It is worth noting that *RPL13a* remains highly stable across four conditions: different embryonic developmental stages, different ovarian developmental stages, tissues from both males and females, and following infection with white spot syndrome virus (WSSV). These findings contribute to the selecting and validating reference genes for qPCR analyses in *Procambarus clarkii* under the specific experimental conditions evaluated in this study.

## Simple Summary

The reliability of real-time quantitative PCR (qPCR) results depends on the stable expression of reference genes. However, suitable reference genes for *Procambarus clarkii* under non-ionic ammonia (NH_3_) stress have not been sufficiently evaluated. In this study, the expression stability of 13 candidate reference genes under NH_3_ stress was systematically evaluated by integrating transcriptome screening and literature reports. *RPL13a*, *RPS18*, and *EIF4A* showed the highest overall stability under NH_3_ stress. It is particularly important that *RPL13a* also showed relatively stable expression across the embryonic developmental stages, ovarian developmental stages, male and female tissue panels, and WSSV-infection conditions examined in this study. These findings contribute to the selection of reference genes for qPCR analyses in *Procambarus clarkii* under the specific experimental conditions evaluated in this study.

## 1. Introduction

Gene expression in organisms is profoundly regulated by environmental stimuli, involving complex transcriptional and translational regulatory mechanisms [1,2,3,4]. Compared to protein-level detection, transcriptomic analysis is more cost-effective and convenient, and has become the primary approach for studying gene expression [2,5]. Real-time quantitative PCR (qPCR) has become the method of choice for gene expression analysis due to its high specificity, sensitivity, accuracy, and cost-effectiveness [6,7,8]. However, qPCR is susceptible to various factors, such as template quality and primer specificity. Therefore, the introduction of reference genes for data normalization is essential to mitigate or eliminate systematic errors between samples [9,10,11]. Accordingly, the rational selection of reference genes is an essential prerequisite for ensuring the accuracy and reliability of qPCR analysis [12]. Ideally, reference genes should be stably expressed and remain unaffected by tissue type, developmental stage, sex, or environmental stress. Currently, genes such as beta-actin (*β-actin*), glyceraldehyde-3-phosphate dehydrogenase (*GAPDH*), *18S ribosomal RNA* (*18S rRNA*), and elongation factor 1-alpha (*EF-1α*) are widely used as reference genes in gene expression studies of crustaceans [13,14]. However, accumulating evidence indicates that the expression of certain traditional reference genes may vary to different extents across species or under varying experimental conditions. The direct use of unverified reference genes can compromise the accuracy and reliability of qPCR results [15,16,17,18]. Therefore, systematic screening and validation of appropriate reference genes tailored to specific experimental conditions remain a critical prerequisite for reliable qPCR analysis.

With the advancement of transcriptomic sequencing (RNA-seq) technology, screening candidate internal reference genes based on transcriptomic expression profiles has become a key strategy for improving the efficiency of internal reference gene selection. RNA-seq enables the systematic assessment of the expression abundance and variation patterns of a large number of genes across different samples, thereby identifying candidate genes with moderate expression levels and high stability; however, the screening results still require validation using qPCR and various algorithms. This strategy has been applied to the identification and evaluation of internal reference genes in various aquatic animals, such as *Danio rerio*, *Sebastes schlegelii*, *Mizuhopecten yessoensis*, and *Lutjanus erythropterus* [19,20,21,22]. Meanwhile, various statistical models have been developed for the systematic evaluation of reference gene stability. Specifically, geNorm determines the optimal reference genes by calculating the gene expression stability measure (M value) [23]; NormFinder estimates the expression stability of each gene by evaluating intra- and inter-group variations [24]; and BestKeeper directly compares the expression variability of candidate genes based on the standard deviation (SD) and coefficient of variation (CV) of Ct values [25], and the comparative Delta Ct method, which ranks candidate genes according to the standard deviation (SD) of the pairwise ΔCt values between genes within each sample, on the premise that the ΔCt between two stably expressed genes remains constant across samples. Given the distinct advantages of each algorithm, their combined application, supplemented by the weighted comprehensive ranking provided by the RefFinder platform, can more objectively reflect the true stability of reference genes.

*Procambarus clarkii* has become one of the important aquaculture species in China, ranking fourth in China’s freshwater aquaculture species [26,27,28,29,30]. However, with the promotion of intensive aquaculture mode, the decomposition of residual bait, feces and organic matter leads to the continuous accumulation of ammonia nitrogen in aquaculture water. Due to the unique benthic and burrowing habits of *Procambarus clarkii*, it is exposed to high concentrations of ammonia nitrogen at the bottom of the pond for a long time, and its anaerobic decomposition further aggravates local ammonia nitrogen enrichment [31,32]. Ammonia nitrogen exists in water in two forms: ionic ammonia (NH_4_+) and non-ionic ammonia (NH_3_). NH_3_ is uncharged, easily penetrates biological membranes, and is the primary form responsible for the toxicity of ammonia nitrogen [27,33,34]. The toxicity of NH_3_ is highly environment-dependent and is influenced by pH and temperature [35]. Particularly during the peak aquaculture season, rising water temperatures and improper feeding management can cause pH levels to rise, leading to a sharp increase in the NH_3_ ratio and further exacerbating the toxic effects of ammonia nitrogen [36]. It remains unclear exactly which molecular regulatory networks mediate the toxic effects of NH_3_ in *Procambarus clarkii*. To elucidate this mechanism, it is necessary to systematically monitor the dynamic changes in the expression of relevant genes at the transcriptional level, which is highly dependent on the availability of rigorously validated stable reference genes under NH_3_ stress. Genes such as *β-actin*, *GAPDH*, and *EF-1α* are commonly used as reference gene in crustaceans; however, their suitability can be significantly affected by experimental conditions [37,38]. More importantly, the expression stability of a given reference gene often varies considerably across different tissues, developmental stages, and stress conditions [39]. Therefore, screening for and validating specific reference genes suitable for NH_3_ stress conditions is crucial for elucidating the response mechanisms.

In this study, we first confirmed the toxic effects of NH_3_ on *Procambarus clarkii* through histopathological observations. Subsequently, by integrating transcriptomic sequencing data with a systematic evaluation using multiple algorithms (geNorm, NormFinder, BestKeeper, Delta CT, RefFinder), we screened for and validated stable reference genes suitable for use under NH_3_ stress conditions. More importantly, we further validated the broad applicability of the candidate genes across various contexts, including embryonic development, ovarian development, tissues from both male and female individuals, and viral infection. This study aims to identify a highly stable reference gene in the *Procambarus clarkii*, which will not only provide a reliable standardization tool for gene expression studies under NH_3_ stress but will also serve as a reference gene for research on embryonic development, ovarian development, sex differences, and viral infections.

## 2. Materials and Methods

### 2.1. Procambarus clarkii

*Procambarus clarkii* were collected from East Dongting Lake and maintained at the Hunan Fisheries Research Institute and Aquatic Products Seed Stock Station, Changsha, China.

### 2.2. Ethics Statement

This study was approved by the HUNAU Animal Care and Use Committee (Granted number 2024EA126). All of the experimental procedures involving animals were conducted in accordance with the rule of Care and Use of Laboratory Animals in China, Animal Ethical and Welfare Committee of China Experimental Animal Society.

### 2.3. Acute Toxicity Test for Non-Ionic Ammonia (NH_3_)

Properly active Procambarus clarkii weighing 8.76 ± 0.89 g were selected for the experiment; feeding was discontinued 24 h prior to the experiment. A control group and six treatment groups with different concentrations of NH3 were established, with concentrations of 0.6, 0.9, 1.2, 1.5, 1.8, and 2.1 mg/L, respectively. The corresponding NH4Cl additions, after conversion, were equivalent to total ammonia nitrogen concentrations of 100, 150, 200, 250, 300, and 350 mg/L, respectively. NH_4_Cl was used as the ammonia source. TAN was defined as the sum of non-ionic ammonia nitrogen (NH_3_-N) and ionized ammonium nitrogen (NH_4_^+^-N). The NH_4_Cl powder used in the experiment (purity ≥ 99.5%) was dried in an oven at 105 °C for 2 h until constant weight was reached. After cooling, the required amount was weighed out and adjusted to the target concentration. Before the exposure experiment, preliminary solution-preparation tests were conducted to verify the nominal TAN concentrations. TAN was measured using a TE-1800 portable multiparameter water-quality analyzer (Tianer Analytical Instrument (Tianjin) Co., Ltd., Tianjin, China), and the measured concentrations were close to the corresponding nominal values. Therefore, the exposure solutions used in the subsequent experiments were prepared according to the nominal TAN concentrations. The fraction of un-ionized ammonia was calculated from water temperature and pH according to the ammonia-equilibrium equations described by Emerson et al. [40]. During the exposure experiment, water temperature and pH were measured daily and maintained at approximately 23 °C and 7.0, respectively. Fresh NH_4_Cl exposure solutions corresponding to the designated nominal TAN concentrations were prepared daily and used for water renewal to maintain the target exposure conditions [40]. Each group consisted of three independent tanks, with 20 shrimp randomly assigned to each tank. Each tank was considered an independent experimental unit.

### 2.4. White Spot Syndrome Virus (WSSV) Infection Experiment

Prior to the experiment, *Procambarus clarkii* individuals were screened via qPCR to ensure they were free of WSSV. The WSSV inoculum was derived from the hepatopancreas tissues of naturally infected *Procambarus clarkii.* Following quantification of the viral copy number using real-time quantitative PCR, a viral tissue homogenate suspension was prepared in PBS buffer (pH 7.4) for subsequent use. Healthy *Procambarus clarkii* (12–15 g per individual) with intact carapaces, reared under high-density and high-temperature conditions, were randomly divided into a control group and a WSSV infection group, with three replicates per group. For the infection group, the viral tissue homogenate suspension was added to the rearing water to a final concentration of 2.14 × 10^3^ copies/mL. The shrimp were immersed in this suspension for 5 min at a density of 35–40 individuals/L, and then transferred to virus-free natural water for continuous rearing. Mortality was monitored daily, and dead individuals were promptly removed. At 10 days post-infection, eight tissues (gonad, intestine, antennal gland, hepatopancreas, heart, muscle, gill, and eye) were collected from each group, immediately snap-frozen in iquid nitrogen, and stored for subsequent RNA extraction [13,41,42].

### 2.5. Sample Collection

The samples collected in this study were categorized into five groups: (1) Eight tissues (gonad, intestine, antennal gland, hepatopancreas, heart, muscle, gill, and eye) from both the 96 h NH_3_ stress group and the corresponding control group. For each tissue type, tissues collected from three crayfish randomly selected from each tank were pooled to form one biological replicate. Thus, three independent pooled biological replicates were obtained for each treatment group. (2) Embryos at different developmental stages (cleavage stage, blastula stage, gastrula stage, early nauplius stage, late nauplius stage, compound eye pigmentation stage, pre-hatching stage, and hatching stage), which were staged via microscopic examination prior to collection. Three independent biological replicates were prepared at each developmental stage, with each replicate consisting of a pooled sample of 10–15 embryos. (3) Ovaries at different developmental stages (stages I–VI), which were histologically identified by HE staining prior to collection. Three independent biological replicates were prepared at each developmental stage, with each replicate consisting of ovarian tissues pooled from three female crayfish. (4) Eight tissues (as listed above) were collected from male and female crayfish. For each tissue type, tissues collected from three crayfish of the same sex were pooled to form one biological replicate. Three independent pooled biological replicates were prepared for each sex. (5) Eight tissues (as listed above) from both the white spot syndrome virus (WSSV) infection group and the control group. For each tissue type, tissues collected from three crayfish within the same experimental replicate were pooled to form one biological replicate. Three independent pooled biological replicates were included per group. Upon dissection, all tissue samples were immediately snap-frozen in liquid nitrogen and subsequently stored at −80 °C until further use. Samples intended for histopathological observation were fixed in 4% paraformaldehyde solution.

### 2.6. Hematoxylin and Eosin (HE) Staining

Following exposure to different concentrations of NH_3_ for 48 h, gill, hepatopancreas, and muscle tissues were collected from each group for histopathological examination. Simultaneously, ovarian tissues from *Procambarus clarkii* at different developmental stages were collected. All tissue samples were fixed in 4% paraformaldehyde for 24 h, followed by standard stepwise ethanol dehydration, xylene clearing, and paraffin embedding. After deparaffinization with xylene and rehydration with a gradient of ethanol, the sections were stained with hematoxylin and eosin (HE), followed by dehydration with a gradient of ethanol, clearing with xylene, and finally mounted with neutral resin. The stained sections were examined and photographed using an Olympus BX53 light microscope (Olympus Corporation, Tokyo, Japan).

### 2.7. Transcriptome Sequencing and Analysis

Transcriptomic sequencing was performed on the hepatopancreas tissues of *Procambarus clarkii* from the 96 h NH_3_ stress group and the control group, with three biological replicates per group. Total RNA purity and concentration were assessed using a NanoDrop 2000 spectrophotometer (Thermo Fisher Scientific, Waltham, MA, USA), and RNA integrity was evaluated using an Agilent 2100 Bioanalyzer (Agilent Technologies, Santa Clara, CA, USA). For library construction, mRNA was enriched using oligo (dT)-containing magnetic beads and fragmented. First- and second-strand cDNA were then synthesized, and the resulting libraries were purified using AMPure XP beads (Beckman Coulter, Inc., Brea, CA, USA). Library concentration and fragment-size distribution were assessed using a Qubit 3.0 fluorometer (Life Technologies Holdings Pte Ltd., Singapore) and a Qsep400 system (Bioptic Inc., New Taipei City, Taiwan), respectively. Qualified libraries were sequenced in paired-end 150 bp mode on a high-throughput sequencing platform. After adapter removal and quality filtering, the resulting clean reads were aligned to a custom *Procambarus clarkii* reference genome assembly using HISAT2 (version 2.0.4). The aligned reads were subsequently assembled, and transcript expression levels were quantified using StringTie (version v2.2.1).

Gene-level raw read counts were used for differential expression analysis with DESeq2 (version 1.30.1) [43]. Genes with a fold change ≥ 2 and a false discovery rate (FDR) < 0.01 were considered differentially expressed. The identified differentially expressed genes (DEGs) were subsequently subjected to Gene Ontology (GO) and Kyoto Encyclopedia of Genes and Genomes (KEGG) enrichment analyses. To eliminate background noise, low-abundance transcripts (read counts ≤ 2) were excluded from the dataset. Gene expression levels were measured as the number of fragments per thousand base pairs of transcript per million mapped reads (FPKM) [44]. Higher FPKM values indicate stronger gene expression. In this study, the FPKM values for all genes were transformed using log_2_ (FPKM + 1) [45]. Following the standards established by Li et al. [21] and Han et al. [46], the following four screening criteria were established: (I) Detectable expression in all samples, with FPKM > 0; (II) Low expression variability across samples (standard deviation of log_2_ (FPKM + 1) < 1); (III) The absolute difference between the log_2_ (FPKM + 1) of each sample and the mean log_2_ (FPKM + 1) is <2; (IV) High expression abundance, with mean log_2_ (FPKM + 1) > 5. Additionally, the coefficient of variation (CV), calculated as the standard deviation divided by the mean, was used for each candidate gene. The candidate internal control genes selected based on the above criteria were used for subsequent qPCR validation.

### 2.8. RNA Isolation and cDNA Synthesis

Total RNA was extracted from each biological sample using the FastPure Cell/Tissue Total RNA Isolation Kit V2 (RC112-01, Vazyme, Nanjing, China). Samples included: the NH_3_ stress group (gonads, intestine, antennal glands, hepatopancreas, heart, muscle, gills, and eyes from both control and 96 h post-stress individuals); embryos at different developmental stages (cleavage stage, blastula stage, gastrula stage, early nauplius stage, late nauplius stage, compound eye pigmentation stage, pre-hatching stage, and hatching stage); ovaries at different developmental stages (stages I–VI); eight tissue types each from male and female individuals (gonads, intestine, antennal glands, hepatopancreas, heart, muscle, gills, and eyes), as well as eight tissue types each from the white spot syndrome virus (WSSV)-infected group and the control group. RNA quality was assessed using 1% agarose gel electrophoresis and a nucleic acid analyzer. Subsequently, 1 μg of total RNA was used for cDNA synthesis via reverse transcription using the HiScript III 1st Strand cDNA Synthesis Kit (R223-01, Vazyme), following the kit instructions. The resulting cDNA was stored at −20 °C for future use.

### 2.9. Real-Time Quantitative PCR (qPCR)

The qPCR primers were designed using the Olig7 software 7.60 and were listed in Table 1. The specificity of the primers was verified by RT-PCR and NCBI blast (https://blast.ncbi.nlm.nih.gov/Blast.cgi, accessed on 11 October 2026) was used to ensure that the sequence similarity was above 99% before applying to qPCR. For qPCR, each 10 µL qPCR reaction mixture contains 1 µL of cDNA, 5 µL of ChamQ Universal SYBR qPCR Master Mix (Q712, Vazyme), 0.5 µL each of forward and reverse primers at a final concentration of 0.5 µM, and 3 µL of DEPC water. Three independent biological replicates were analyzed for each sample and experimental condition. A no-template control (NTC), in which nuclease-free water was used instead of the cDNA template, was included in each assay to monitor potential reagent contamination and nonspecific amplification [47,48,49]. The entire reaction was conducted using Roche LightCycler^®^96 real-time quantitative PCR system (Roche, Basel, Switzerland). The specificity of all qPCR was confirmed by melting curve analysis of amplification products (Figure S2) [50]. Amplification efficiency and standard-curve correlation coefficients were determined using a serially diluted pooled cDNA sample; only primers with amplification efficiencies within 90–110% and correlation coefficients (R^2^) above 0.97 were used for subsequent analyses. The Ct values obtained for all candidate reference genes across different samples were exported for subsequent analyses, with lower Ct values indicating higher relative mRNA transcript abundance [51].

### 2.10. Evaluation of Gene Expression Stability

qPCR was used to obtain Ct values for each candidate reference gene across different samples. The expression stability of each candidate gene was systematically evaluated using the mainstream algorithms geNorm, NormFinder, and BestKeeper. geNorm measures the stability of a reference gene by calculating the gene stability value (M); a lower M value indicates higher stability. The optimal number of reference genes was further assessed using the geNorm pairwise variation values (Vn/Vn+1), which compare normalization factors calculated using n and n + 1 reference genes. A Vn/Vn+1 value below 0.15 was used as a commonly applied reference for determining whether the addition of another reference gene was necessary. NormFinder evaluates the stability of each gene by assessing intra- and inter-group variability. BestKeeper analyzes the standard deviation (SD) and coefficient of variation (CV) of Ct values to directly compare the expression variability of candidate genes. The comparative ΔCt method ranks the candidate genes according to the standard deviation (SD) of the pairwise ΔCt values between genes within each sample, on the premise that the ΔCt between two stably expressed genes remains constant across samples; a smaller SD of ΔCt indicates higher and more consistent expression stability. RefFinder calculates a weighted geometric mean to derive a comprehensive stability ranking for each candidate gene [12]. A lower stability value indicates more stable gene expression.

## 3. Results

### 3.1. Histopathological Alterations in Different Tissues Following NH_3_ Exposure

**Figure 1:**
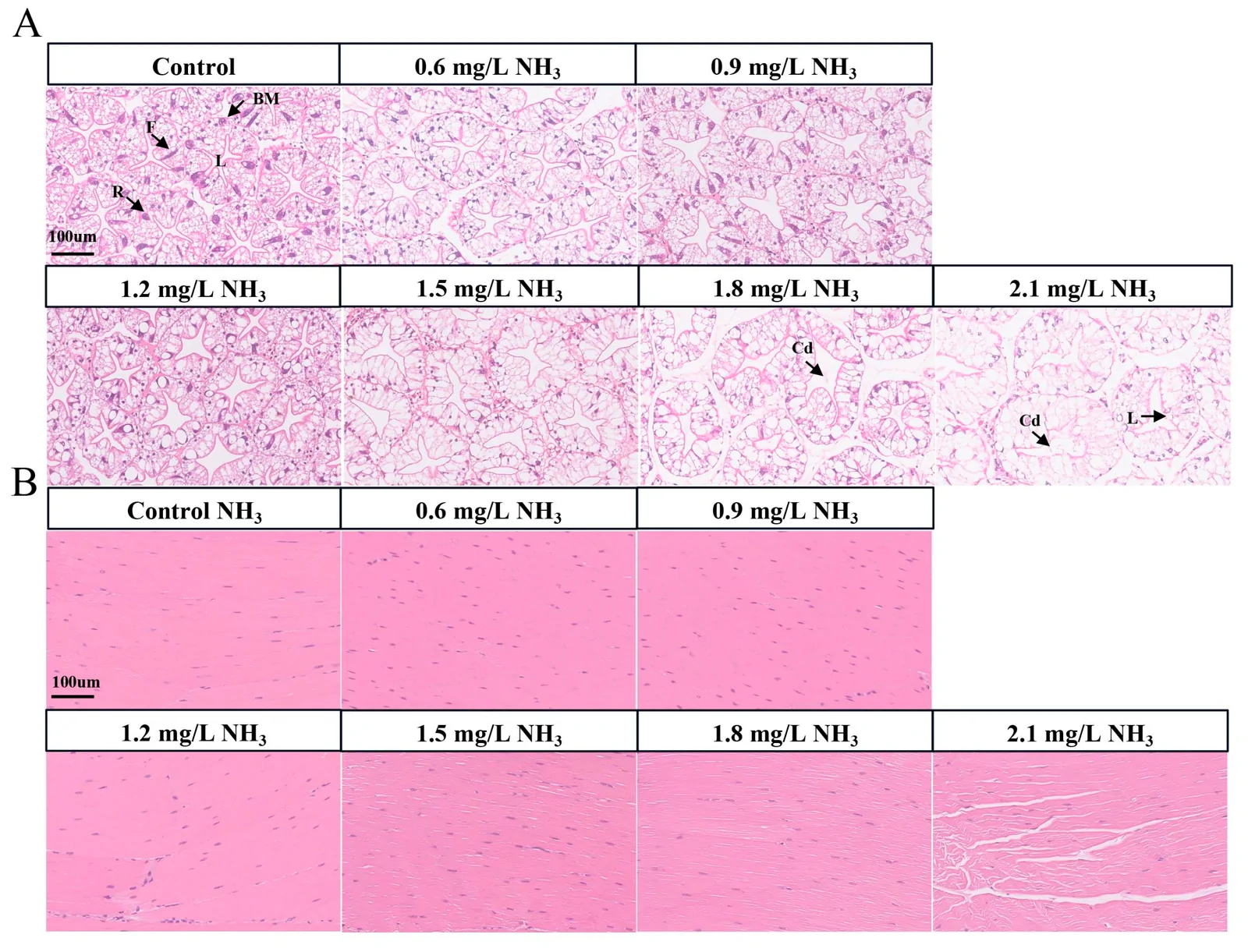
Effects of NH_3_ stress for 48h on histopathology of hepatopancreas and muscle tissue. (**A**) Hepatopancreas. (**B**) Muscle. R: Absorptive cell; F: Fibrillar cell; L: Hepatopancreatic tubule lumen; BM: Basement membrane; Cd: Cell debris. Scale bar = 100 μm.

To investigate the toxic effects of non-ionic ammonia (NH_3_) on *Procambarus clarkii*, the changes of gill, hepatopancreas, and muscle tissues after 48 h of NH_3_ stress at different concentrations were observed by HE staining. Histopathological alterations of increasing apparent severity were observed with increasing NH_3_ concentrations. (Figure S1 and Figure 1). In the gill tissue, the gill tissue structure of the control group was clear and complete, the stratum corneum was thin and continuous, the gill epithelial cells were closely arranged, and the respiratory epithelial cells were closely attached to the stratum corneum without obvious separation. In the 0.6–1.2 mg/L treatment group, the morphology of gill tissue was basically normal, and only some respiratory epithelial cells were slightly separated from the stratum corneum. In the 1.8 mg/L and 2.1 mg/L treatment groups, serious damage occurred, respiratory epithelial cells were significantly separated from the stratum corneum, some gill lamellae collapsed, and a large number of cell debris were observed (Figure S1).

In the hepatopancreas, the structure of the control group was intact, the morphology of the hepatic tubular lumen (L) was regular, and the basement membrane (BM) was clear. With the increase in NH_3_ concentration, tissue damage was aggravated in a dose-dependent manner. In the 0.6 mg/L–1.2 mg/L treatment group, the lumen of the hepatic tubules was slightly dilated, and the basement membrane remained relatively intact. In the 1.8 mg/L and 2.1 mg/L treatment groups, the hepatic tubules were ruptured, the basement membrane was severely broken, a large amount of cell debris (Cd) was accumulated in the lumen, and the absorbing cells (R) were extensively disintegrated and necrotic (Figure 1A). In the muscle tissue, the muscle fibers in the control group were arranged neatly and tightly, and the perimysium was intact. In the 1.5 mg/L treatment group, only part of the muscle fiber gap was slightly widened; in the 2.1 mg/L treatment group, the muscle fibers were extensively broken and dissolved, the arrangement was seriously disordered, the stripes disappeared, the perimysium was broken, and muscle fiber fragments and cavities appeared in some areas. The above results indicate that NH_3_ stress can cause dose-dependent pathological damage to gill, hepatopancreas and muscle tissues of *Procambarus clarkii* (Figure 1B).

### 3.2. Transcriptional Response Characteristics of the Hepatopancreas Under Non-Ionized Ammonia Stress

**Figure 2:**
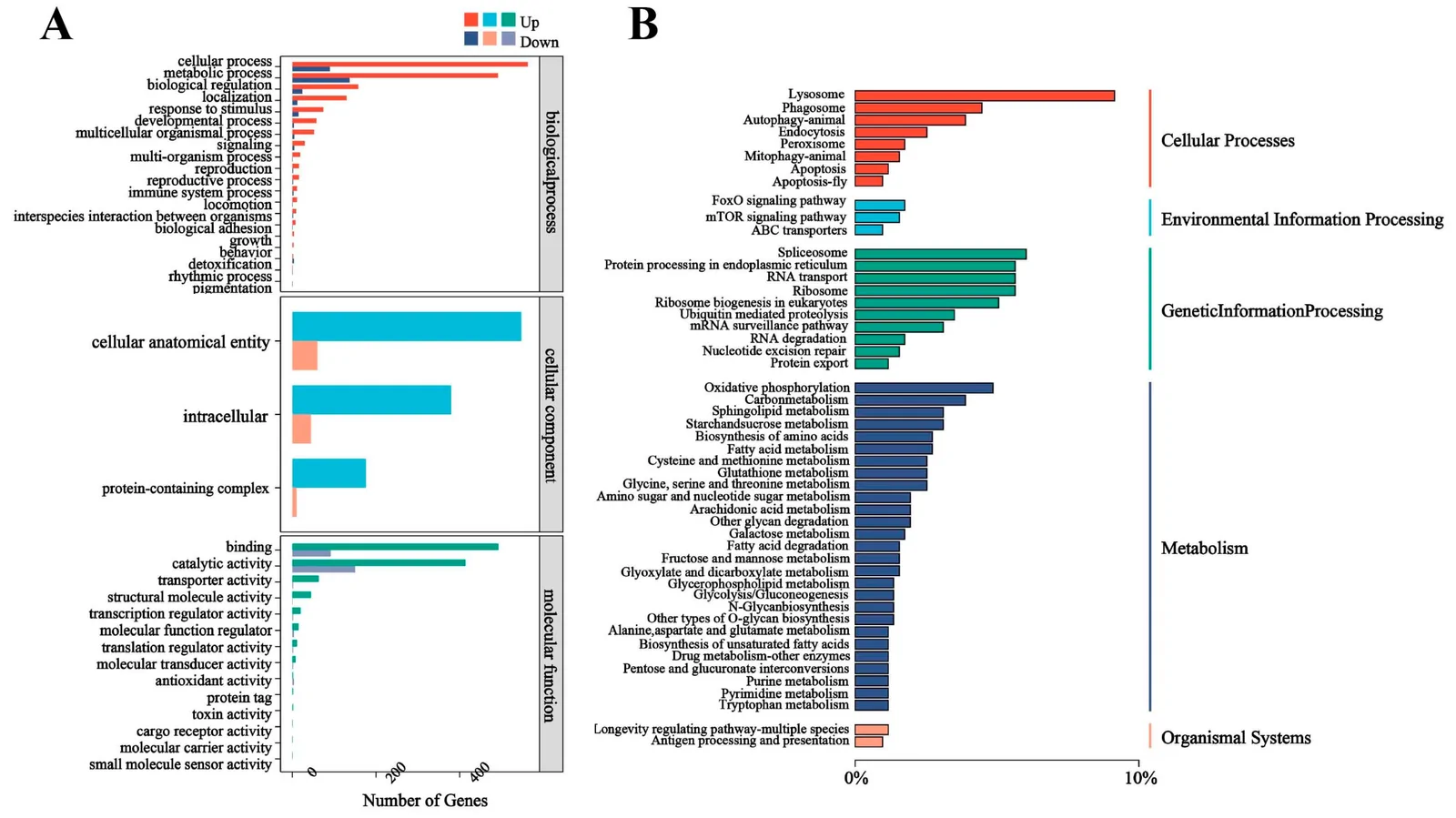
GO and KEGG enrichment analysis of differentially expressed genes in the hepatopancreas under NH_3_ stress. (**A**) GO category plot. (**B**) KEGG category plot.

Hepatopancreas is the main metabolic and detoxification organ of crustaceans, and transcriptome sequencing was performed on hepatopancreas. The results showed that GO enrichment analysis showed that differentially expressed genes were significantly enriched in biological processes such as “metabolic process”, “cellular process” and “stimulus response”, especially in molecular functional categories such as “oxidoreductase activity”and “transporter activity” (Figure 2A); KEGG pathway enrichment analysis further revealed that the differentially expressed genes were mainly focused on key signaling pathways such as energy metabolism and lysosomes (Figure 2B). It is suggested that NH_3_ stress interferes with the normal metabolic homeostasis of the body and induces a strong stress response. Traditional reference genes may have expression fluctuations, and it is necessary to identify stable reference genes suitable for this specific experimental condition through systematic screening.

### 3.3. Screening of Candidate Reference Genes Based on Transcriptomic Data

**Figure 3:**
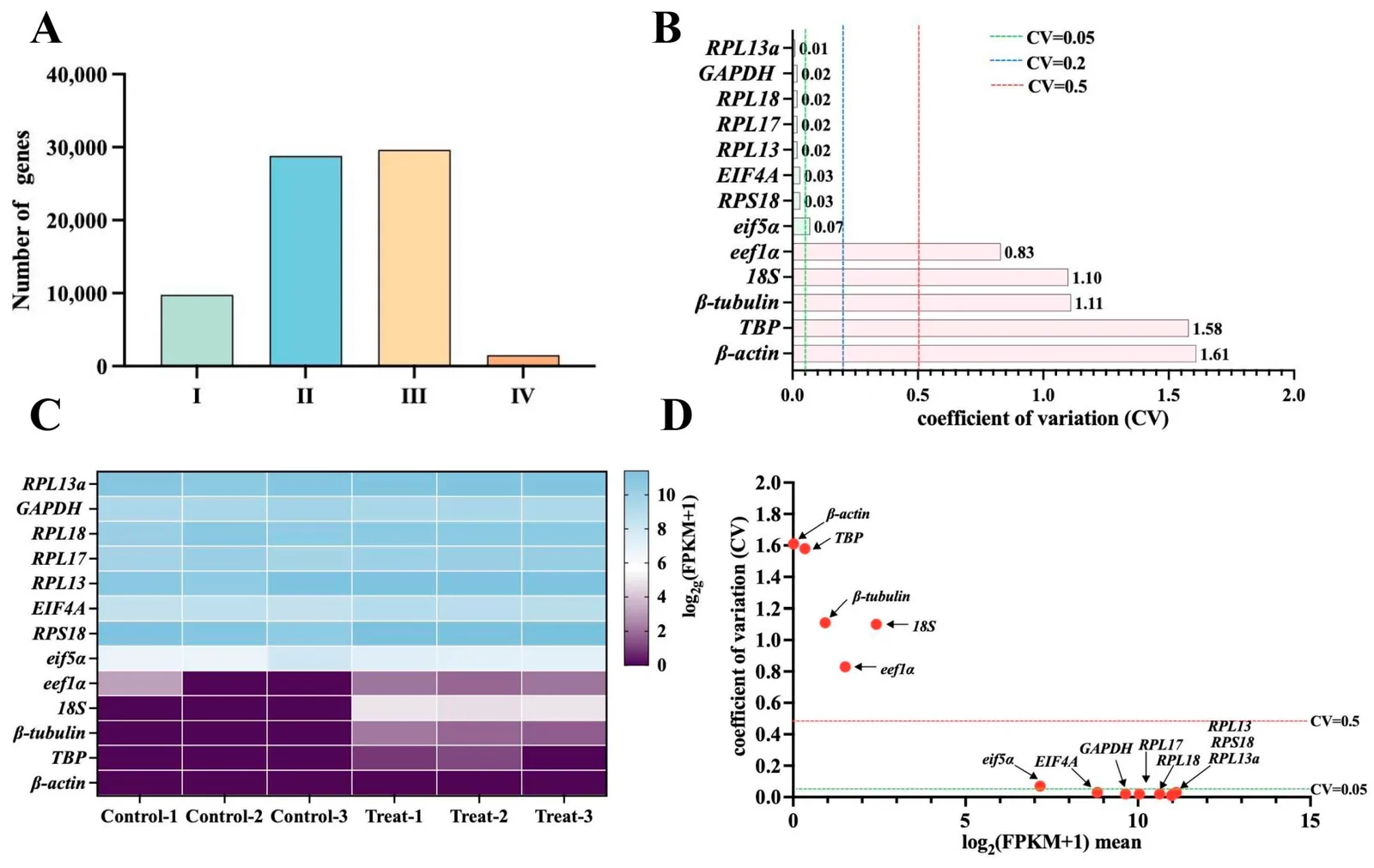
Screening and expression stability evaluation of candidate reference genes in *Procambarus clarkii* based on transcriptomic data. (**A**) The number of genes retained after applying four screening criteria to the *Procambarus clarkii* transcriptomic data: (I) FPKM > 0; (II) standard deviation of log_2_ (FPKM + 1) < 1; (III) difference between log_2_ (FPKM + 1) and mean log_2_ (FPKM + 1) <2; (IV) mean log_2_ (FPKM + 1) > 5. A total of 1271 genes simultaneously met all the above criteria. (**B**) Comparison of the coefficient of variation (CV) among the 13 candidate reference genes. (**C**) Heatmap showing the expression levels of the 13 candidate reference genes across six samples from the control and NH_3_ treatment groups. (**D**) Scatter plot showing the relationship between the mean expression level log_2_ (FPKM + 1) and the CV of the candidate reference genes. The dashed lines represent the CV thresholds of 0.05, 0.2, and 0.5. Genes located in the lower right corner and below the threshold lines indicate higher expression stability and moderate abundance.

To identify potential reference genes, transcriptomic sequencing was performed on hepatopancreatic tissues following non-ionic ammonia stress. The raw RNA-seq data generated in this study will be made publicly available in the Genome Sequence Archive (GSA) at the National Genomics Data Center (NGDC) under project accession number PRJCA071042. A total of 29,897 genes in the RNA-seq database were systematically screened based on four criteria: (I) gene expression in the samples FPKM > 0); (II) low expression variation (standard deviation of log_2_ (FPKM + 1) < 1); (III) a difference of less than 2 between log_2_ (FPKM + 1) and the mean log_2_ (FPKM + 1); and (IV) high expression abundance (mean log_2_ (FPKM + 1) > 5). As shown in Figure 3A, 9784 (32.73%), 28,802 (96.34%), 29,647 (99.16%), and 1492 (4.99%) genes met these criteria, respectively. Ultimately, 1271 genes simultaneously satisfied all four criteria and were identified as potential stably expressed genes. 13 candidate reference genes were ultimately selected for validation, comprising 8 highly stable genes identified from the transcriptomic data (*GAPDH*, *RPS18*, *RPL17*, *RPL18*, *eif5α*, *RPL13*, *EIF4A*, and *RPL13a*) and 5 commonly used reference genes reported in previous literature (*β-actin*, *β-tubulin*, *18S*, *TBP*, and *eef1α*) (Table S1) [13,52,53]. The coefficient of variation (CV) analysis revealed that 8 genes (*RPL13a*, *GAPDH*, *RPL18*, *RPL17*, *RPL13*, *EIF4A*, *RPS18*, and *eif5α*) exhibited CV values less than 0.2, with *RPL13a* showing the lowest CV value. In contrast, the traditional reference genes *β-actin* and *β-tubulin* displayed CV values greater than 0.5, preliminarily indicating that their expression stabilities were compromised under NH_3_ stress (Figure 3B). The heatmap demonstrated that *RPL13a* maintained a uniform expression level across all samples, whereas *eef1α* and *β-tubulin* exhibited significant expression fluctuations (Figure 3C). Furthermore, the scatter plot of expression level versus CV indicated that *RPL13a* and *RPS18* possessed both low CV values and high expression abundances, making them ideal candidate reference genes (Figure 3D).

### 3.4. Variation Range of Ct Values for Candidate Reference Genes in Eight Different Tissues Under NH_3_ Stress

**Figure 4:**
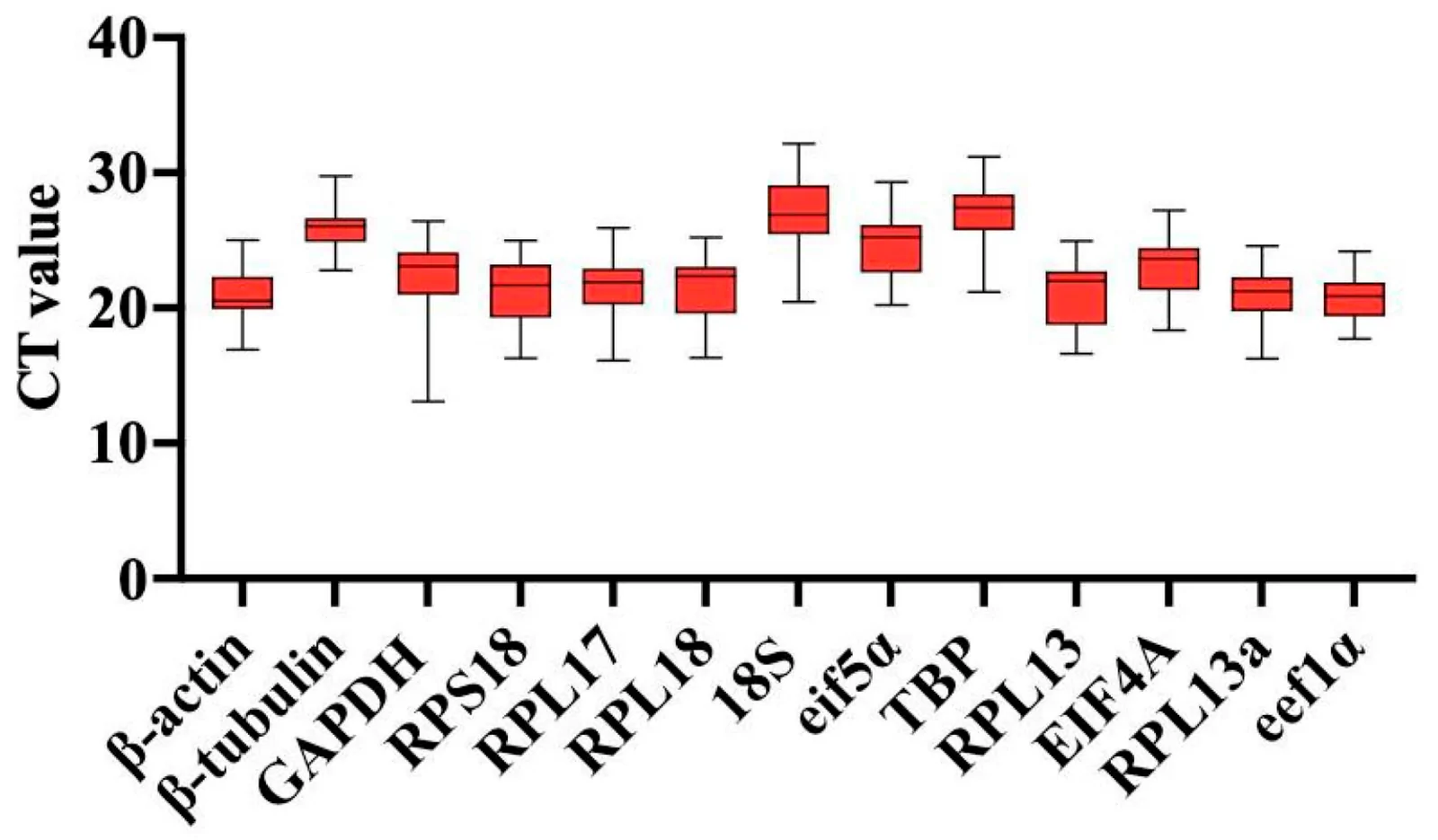
Variation range of Ct values for candidate reference genes in eight different tissues of *Procambarus clarkii* under NH_3_ stress.

To verify the expression levels and variability of the candidate reference genes in different tissues, the Ct values of the 13 candidate genes were determined by qPCR in eight tissue types (gonad, intestine, antennal gland, hepatopancreas, heart, muscle, gills, and eye) of *Procambarus clarkii* following NH_3_ stress. The qPCR primers for the candidate reference genes are shown in Table 1. The correlation coefficients of the standard curves for all primers were greater than 0.97, and the amplification efficiency of all primers was above 94% (Table S2). Furthermore, the melting curves exhibited single peaks with no artifacts, indicating good primer specificity and single amplification products (Figure S2). The Ct values obtained from the baseline threshold of qPCR were used to measure the expression abundance of reference genes; Ct values are inversely proportional to gene expression abundance. Box plots show the expression levels of the candidate genes across different tissue samples. The Ct values for the vast majority of candidate reference genes ranged from 16 to 26 across different tissue samples, indicating high expression levels. Among them, *eef1α* (17.71–24.18), *β-tubulin* (22.78–29.75), *β-actin* (16.91–25.03) and *RPL13a* (16.26–24.58) exhibited relatively narrow Ct ranges. In contrast, *GAPDH* (13.09–26.42), *18s* (20.46–32.16), *TBP* (21.19–31.17) and *eif5α* (20.23–29.32) exhibited relatively wide ranges of Ct values (Figure 4).

### 3.5. Comprehensive Evaluation and Validation of Reference-Gene Stability in Fluorescence Quantitative PCR Under NH_3_ Stress

**Figure 5:**
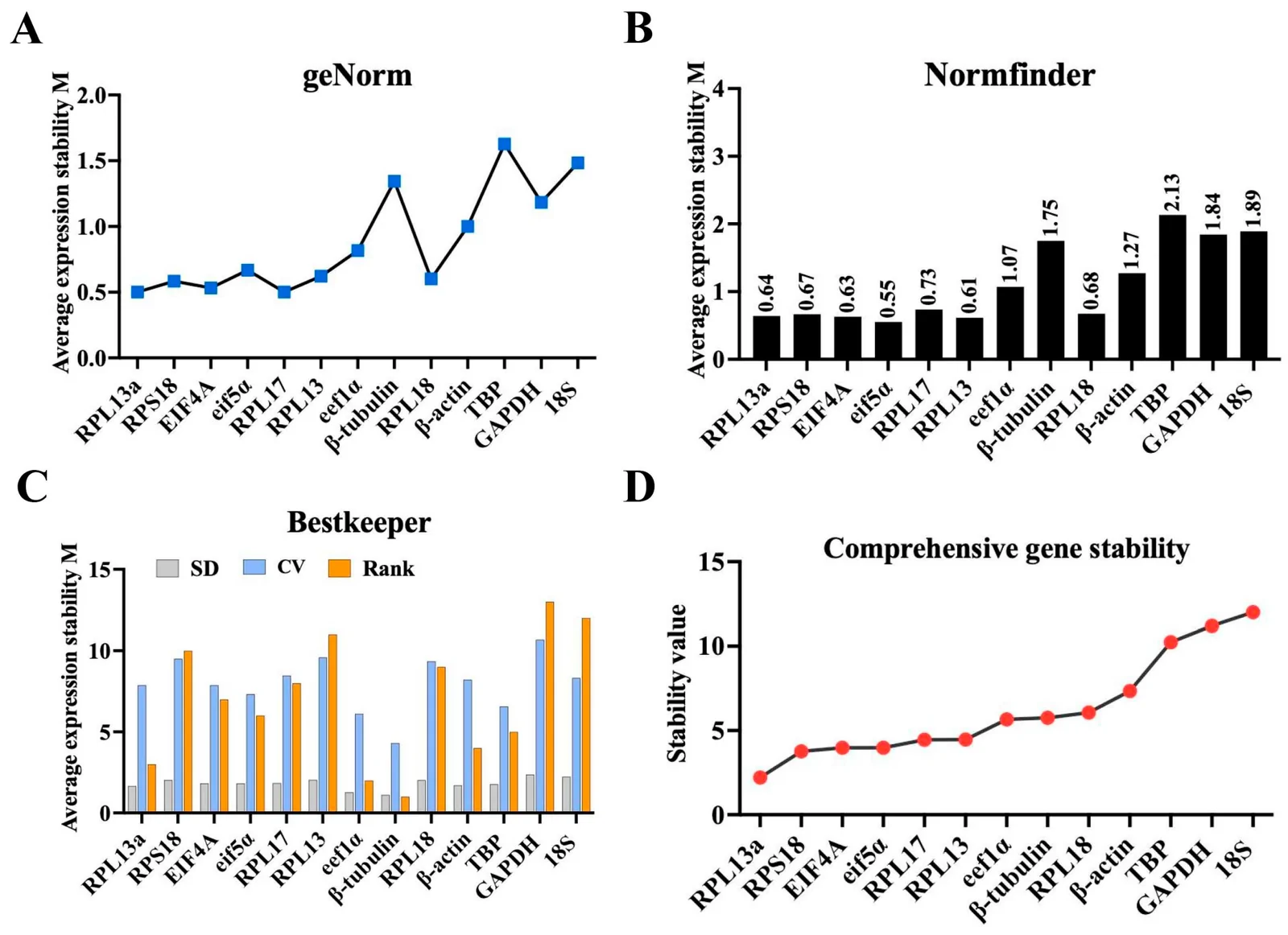
Evaluation of the expression stability of candidate reference genes in eight tissues of *Procambarus clarkii* under NH_3_ stress. (**A**) geNorm evaluates stability. (**B**) Normfinder evaluates stability. (**C**) Bestkeeper evaluates stability. (**D**) Comprehensive ranking of candidate reference genes based on stability, calculated using RefFinder’s weighted method.

To systematically evaluate the expression stability of candidate reference genes under NH_3_ stress, four commonly used algorithms geNorm, NormFinder, BestKeeper and Delta CT method were employed, followed by a comprehensive ranking using the RefFinder web tool (https://blooge.cn/RefFinder/#, accessed on 11 October 2026) based on the geometric mean of their respective rankings. The geNorm analysis identified *RPL13a* and *RPL17* as the most stable reference genes, exhibiting the lowest M values (Figure 5A). NormFinder ranked *eif5α* and *RPL13* as the most stable candidates (Figure 5B). BestKeeper, which evaluates stability based on the standard deviation (SD) and coefficient of variation (CV) of Ct values, identified *β-tubulin* as the most stable gene, followed by *eef1α*, and *RPL13a*, all of which displayed relatively low SD values (Figure 5C). The Delta Ct method identified *RPL13a* and *RPS18* as the most stable pair. Due to differences in algorithms, the results from different software vary and a comprehensive ranking was generated using RefFinder. The overall expression stability, ranked from highest to lowest, was determined as follows: *RPL13a*, *RPS18*, *EIF4A*, *eif5α*, *RPL17*, *RPL13*, *eef1α*, *β-tubulin*, *RPL18*, *β-actin*, *TBP*, *GAPDH*, and *18S* (Figure 5D).

Among the individual candidates, *RPL13a* showed the highest overall stability, followed by *RPS18* and *EIF4A*. The geNorm pairwise variation analysis showed that V2/3 was above the commonly used threshold of 0.15, whereas V3/4 decreased to 0.12, suggesting that the inclusion of a third reference gene could further improve normalization under the tested NH_3_-exposure conditions. Based on the integrated stability ranking, *EIF4A* was selected as the third candidate. Further validation using four NH_3_-responsive genes showed that normalization with *RPL13a* + *RPS18* yielded log_2_ fold-change estimates close to those obtained with *RPL13a* + *RPS18* + *EIF4A*. Both combinations produced estimates more consistent with the RNA-seq results than those obtained using *β-actin*, *β-tubulin*, or *GAPDH* (Figure S3). These findings support the use of *RPL13a* + *RPS18* as a practical two-gene normalization strategy under the tested NH_3_ stress, with *EIF4A* serving as an additional reference gene when a three-gene normalization strategy is applied.

### 3.6. The Stability of RPL13a as a Reference Gene Under Different Conditions Was Verified

**Figure 6:**
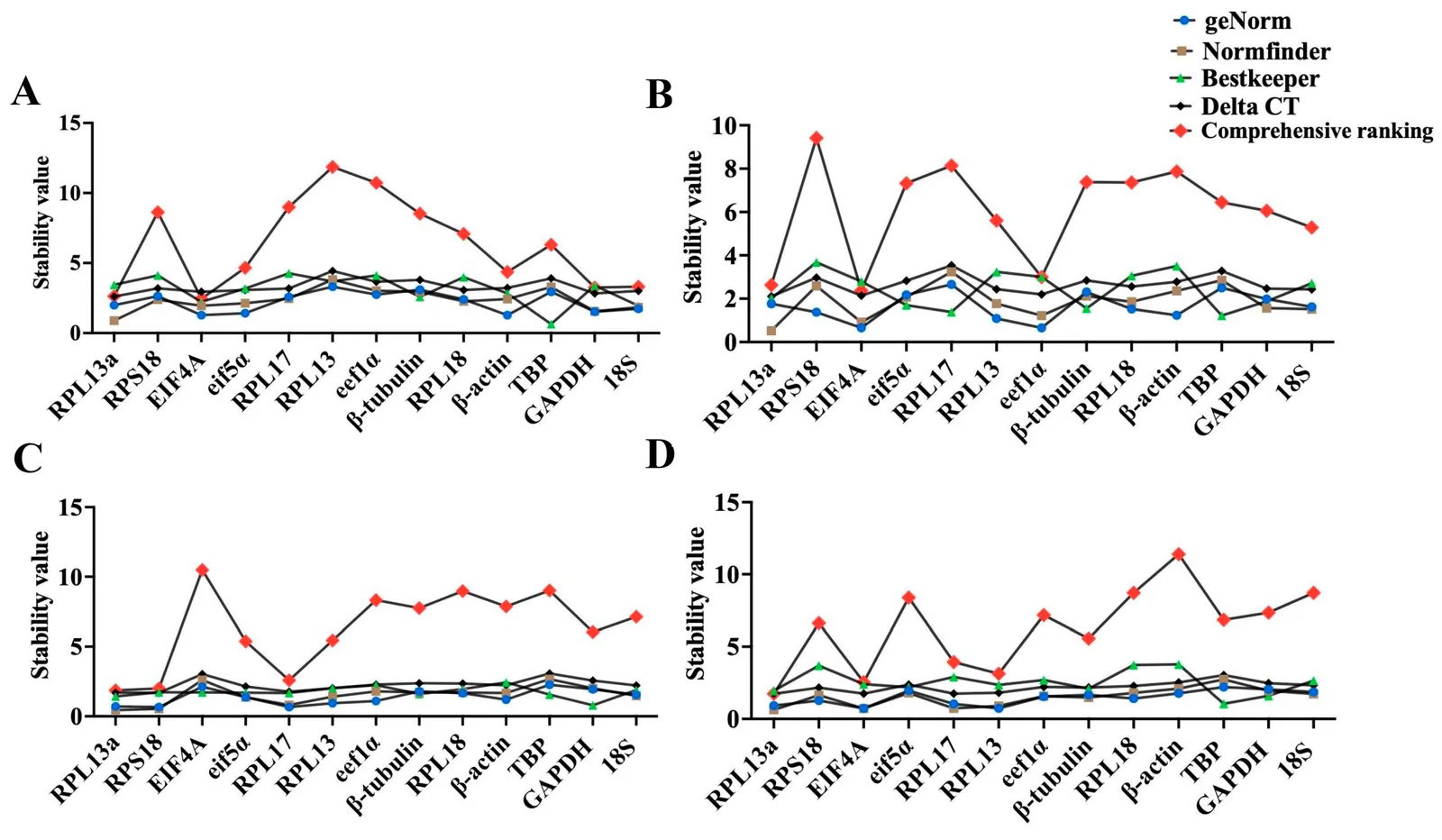
Stability evaluation of *RPL13a* as a reference gene under four different conditions. (**A**) Embryos at eight different developmental stages. (**B**) Ovaries at six different developmental stages. (**C**) Eight different tissues from both male and female individuals. (**D**) Eight different tissues following white spot syndrome virus (WSSV) infection. Lower values indicate higher comprehensive stability.

To investigate whether the selected optimal reference gene is suitable for other conditions, the expression stability of *RPL13a* and other candidate genes was further assessed under four different conditions, including: embryos at eight different developmental stages, ovaries at six different developmental stages, eight tissue types from both male and female individuals, and eight different tissue types following viral (WSSV) infection. The range of Ct values for the 13 candidate reference genes under the four conditions mentioned above is shown in Figure S4. Based on this, the evaluation results from the four algorithms geNorm, NormFinder, BestKeeper and Delta CT were combined and weighted using RefFinder to generate a comprehensive ranking of expression stability, as shown in Figure 6. The results showed that *EIF4A* and *RPL13a* were among the most stable reference-gene candidates across both embryonic and ovarian developmental stages. In tissues of both sexes, *RPL13a* and *RPS18* ranked among the most stable; and following WSSV infection, *RPL13a* and *EIF4A* exhibited the highest stability. Notably, *RPL13a* ranked among the top two most stable genes under all four conditions (Figure 6). These results indicate that *RPL13a* not only remains stable under NH_3_ stress but also maintains good stability during embryonic development, the reproductive cycle, across sex differences, and under pathogen stress conditions.

## 4. Discussion

In this study, *Procambarus clarkii* was used as the research object. Firstly, histopathological examination demonstrated the adverse effects of non-ionic ammonia (NH_3_) on the gill, hepatopancreas, and muscle of *Procambarus clarkii*, indicating that NH_3_ exposure can compromise the structural integrity of multiple tissues. On this basis, the expression stability of 13 candidate genes (*GAPDH*, *RPS18*, *RPL17*, *RPL18*, *eif5α*, *RPL13*, *EIF4A*, *RPL13a*, *β-actin*, *β-tubulin*, *18S*, *TBP* and *eef1α*) in 8 tissues (gonad, intestine, antennal gland, hepatopancreas, heart, muscle, gill, and eye) before and after NH_3_ stress was systematically evaluated based on transcriptome data mining and combined with commonly used reference genes in the literature. The stability of the candidate genes was further examined under the additional experimental conditions evaluated in this study, including embryonic development, ovarian development, sex-related tissue comparisons, and WSSV infection. Among the candidates, *RPL13a* consistently showed relatively high stability across the tested conditions.

It was found that the expression stability of the same reference gene in different tissues was significantly different, which was consistent with the previous reports in polyploid fish (*Cyprinus carpio* and *Carassius auratus*) [54], *Macrobrachium rosenbergii* [55] and *Paralichthys olivaceus* [46], indicating that the stability of the reference gene was tissue and condition-specific and needed to be further verified in different experimental backgrounds. Specifically, in this study, the Ct values of *RPL13a* exhibited the narrowest fluctuation range (16.26–24.58), whereas those of *GAPDH* and *18S* varied across substantially wider ranges (13.09–26.42 and 20.46–32.16, respectively). This indicates that the expression abundances of *GAPDH* and *18S* differ markedly among tissues, which could introduce considerable errors if they were used as reference genes. The comprehensive ranking of multiple algorithms in this study showed that the traditional commonly used reference genes *β-tubulin*, *β-actin* and *GAPDH* were less stable under NH_3_ stress, suggesting that the traditional reference genes without experimental verification may lead to systematic deviation of qPCR data. *β-actin* is involved in the regulation of cytoskeleton, *GAPDH* is involved in glycolysis and redox, and environmental factors also affect its stability [56,57]. The stability of *GAPDH* and *β-tubulin* was the lowest after rainbow trout cells were exposed to triphenyl phosphate (TPhP). The *β-actin* was also only in the middle of the tilapia infection model [58,59]. Therefore, selecting stably expressed reference genes under different experimental conditions is crucial [12]. In the stability assessment, the rankings obtained using different algorithms showed some discrepancies. geNorm focuses on the consistency of expression ratios among candidate genes, NormFinder considers both within-group and between-group variability, while BestKeeper evaluates stability based on the standard deviation and coefficient of variation of the raw Ct values. Therefore, a single algorithm may be influenced by the number of candidate genes, the range of expression levels, and sample heterogeneity, making it difficult to fully reflect true expression stability [56,60,61]. This study employed RefFinder to integrate the weighted results from multiple algorithms. The comprehensive ranking placed *RPL13a*, *RPS18*, and *EIF4A* among the most stable candidates under NH_3_ exposure, with *RPL13a* showing the highest overall ranking. In addition, geNorm pairwise variation analysis yielded a V2/3 value of 0.18 and a V3/4 value of 0.12, indicating that three reference genes were preferable to two for normalization under the tested conditions. Accordingly, *RPL13a*, *RPS18*, and *EIF4A* were recommended as a three-gene combination for qPCR normalization under NH_3_ exposure, while *RPL13a* was retained as the most stable individual candidate. Ribosomal proteins are mainly involved in the basic process of protein synthesis and are generally considered to be less affected by environmental fluctuations. This finding is similar to the results in chickens, and *RPL13* and *RPS18* were confirmed to be the best reference genes in the infection model [62], and *RPS18* was identified among the most stable reference genes in *Macrobrachium rosenbergii* under various experimental settings and in polyploid fish (*Cyprinus carpio* and *Carassius auratus*) [54,55]. Nevertheless, the optimal member of the ribosomal protein family may differ among species and experimental conditions, underscoring the necessity of systematic evaluation within each specific system.

Previous studies on the screening of reference genes were mostly limited to a single experimental condition, resulting in the lack of versatility of the selected reference genes. In this study, *RPL13a* exhibited relatively stable expression across five representative conditions in Procambarus clarkii: NH_3_ stress (environmental stress), embryos at different developmental stages (developmental timing), ovaries at different developmental stages (reproductive cycle), different tissues of male and female (gender dimorphism) and WSSV infection (pathogen stress). It covers the main research fields from developmental timing, reproductive cycle, gender differentiation to environmental and pathogenic stress. The results showed that *RPL13a* was not only stable under NH_3_ stress, but also ranked in the top two under four independent biological backgrounds: different developmental stages of embryos, different developmental stages of ovaries, different tissues of male and female, and WSSV infection. Studies across different species have demonstrated that ribosomal protein-coding genes can serve as suitable reference genes under specific experimental conditions. For instance, *RPL6* in *Japanese flounder* under thermal stress and *RPS18* in the *Macrobrachium rosenbergii* across various experimental settings have both been reported to exhibit acceptable stability [55,63]. However, these findings also suggest that the stability of ribosomal protein genes is not an intrinsic property of the family to which they belong, but rather exhibits gene specificity and condition dependence. In the present study, *RPL13a* showed superior overall stability compared to *RPL17* and *RPL18*, which belong to ribosomal protein family. This observation indicates that a particular member cannot be presumed to be a stable reference gene solely on the basis of its functional annotation as a ribosomal protein; instead, systematic evaluation within the target species and specific experimental system is required. However, unlike other studies, most of the previous studies only screened reference genes suitable for a single specific condition. For example, in the Japanese swamp crayfish, *RPL18* is suitable for adult tissues, while *EIF* is suitable for the embryonic stage. Wang et al. conducted a systematic screening of reference genes across different developmental stages, tissues, and sexes of the Jackfruit borer (*Diaphania caesalis*) and found that different reference genes are required for different developmental stages, tissues, and sexes [64]. The present study identified *RPL13a* as a candidate showing relatively consistent stability across the specific conditions examined in *Procambarus clarkii*, while recognizing that further validation is required under conditions beyond the scope of this study.

The stable expression of *RPL13a* under various conditions may be closely related to its conserved biological functions. Ribosomal proteins play a fundamental role in protein synthesis, and their mRNA levels are typically subject to strict transcriptional regulation to maintain ribosomal homeostasis [65]. However, the stability of different ribosomal protein genes varies. In this study, the Ct value ranges for *RPL18* ranged from 16.32 to 25.21. Although their stability was superior to that of *GAPDH* and *18S*, it was inferior to that of *RPL13a*, indicating that functional differentiation also exists within the same family [66]. As a component of the 60S large subunit of the ribosome, *RPL13a* participates in peptide bond formation-a core catalytic step in protein synthesis [67]. Its expression may be subject to stricter homeostatic regulation, which could explain its ability to maintain stable expression under various conditions. Furthermore, ribosomal protein genes are typically classified as “housekeeping genes”, whose expression levels are relatively unaffected by external environmental disturbances, a characteristic that makes them ideal candidates for internal reference genes [68]. The results of this study are consistent with the above understanding and further support the potential advantages of ribosomal protein family members as reference genes in crustaceans. RPS18 encodes a component of the 40S small subunit that participates in mRNA binding and translation initiation [69]. Notably, *RPS18* expression remained stable in eight tissue types before and after NH_3_ stress; however, its stability was inferior to that of *RPL13a* in four cross-condition validation experiments. The difference in stability between *RPL13a* and *RPS18* may stem from their different localizations within the ribosome (the large subunit versus the small subunit) and the different levels at which their functions are carried out outside the ribosome; the former acts at the protein level, while the latter is activated at the transcriptional level [65,70,71,72]. RPL13a encodes a ribosomal protein involved in protein synthesis, and its expression regulation is relatively independent of transient changes in specific signaling pathways. Overall, *RPL13a* was identified as the most stable individual candidate in the integrated analysis. However, the geNorm pairwise variation analysis indicated that three reference genes were preferable to two under the tested NH_3_-exposure conditions. Accordingly, *RPL13a*, *RPS18*, and *EIF4A* were recommended as a three-gene combination for qPCR normalization. The suitability of this combination, as well as that of *RPL13a* alone, should be further validated when different tissues, treatments, developmental stages, or pathogens are examined.

## 5. Conclusions

This study combined transcriptomic sequencing data to systematically evaluate the expression stability of 13 candidate reference genes in eight different tissues of *Procambarus clarkii* exposed to NH_3_ stress. Based on the comprehensive multi-algorithm ranking, *RPL13a* showed the highest overall stability, followed by *RPS18* and *EIF4A*, whereas the commonly used reference genes *β-actin*, *GAPDH*, and *18S* exhibited relatively low stability. Pairwise variation analysis indicated that including a third reference gene could further improve normalization. Validation using NH_3_-responsive genes showed that (*RPL13a* + *RPS18*) and (*RPL13a* + *RPS18* + *EIF4A*) produced similar expression estimates. These combinations may therefore be used for qPCR normalization under the NH_3_-exposure conditions evaluated in this study. Furthermore, *RPL13a* remained highly stable across four different contexts: various embryonic developmental stages, different ovarian developmental stages, tissues from both males and females, and following infection with white spot syndrome virus (WSSV). Collectively, these findings identify *RPL13a* as a suitable reference-gene candidate for qPCR normalization in *Procambarus clarkii* under the specific experimental conditions evaluated in this study.

## Figures and Tables

**Table 1:** Primer sequences for qPCR analysis.

Gene Name	Forward Primer (5′-3′)	Reverse Primer (5′-3′)	Primer Product (bp)
*β-actin*	TTCCAGCCTTCATTCCTTGGT	CATCCTGTCTGCAATGCCT	156
*β-tubulin*	GACCTCGAACCCGGCACCATG	AGTTCTCCGCCTCCTTCCTCA	181
*GAPDH*	TGTACATGCTGTCACTGCCAC	AATAACCTTTCCGACAGCCT	133
*RPS18*	GTCCAATAACCTCGAGACCAAGC	TCGAACACGGAGACCCCAGA	100
*RPL17*	AGTGTGTTCCTTTCCGTGTC	TACTCTCCGCATTCTTCAGCAG	135
*RPL18*	TTCAGGGTAAACGCAATGCTC	CTTTCAAACTTGCGTCCCTTG	112
*18S*	TATAGCGCATGTAGTACCAG	CTTCTTCCGATTCTGATGGTG	124
*eif5α*	TCATCTTGTTGGTATCGACCT	ATATCATCACGTAAGTCACCAG	171
*TBP*	AACGGCACTTATCTTTTCCTC	CCAACTTCTGTACAATCCGAG	107
*RPL13*	AGAAGGCCAAGAGAATTACCA	TCTTCTGCTGCCTCCTTCG	124
*EIF4A*	GCTCACTGACAAAATGCACCA	TGATCAGAACACGAGACGAAC	122
*RPL13a*	ACATCTCTGGCTCATTCTACCG	GCAACATTCCCCTAACAGTTCG	141
*eef1α*	CCAAAGTACTCAGAGGCTCGAT	ATGTTGTCTCCATTGAAGCCAGA	125
*Cyp2d14*	CCTTCAACCTGGTGCTCT	CACTGTCGCTTTCCTATGCC	232
*Ptprq*	GACAGCGACTTCTCCAACGA	TGTATCAGCCGTATAACCAGT	177
*Gba*	CAATGGCATGTTCAACGGAT	AATTATCGAGGCCACCAAGC	155
*PclaChr8G028860*	TGACGACCTGATGAACGACT	CACGTAGAATTTACCAGGACGA	154

## Data Availability

Data will be made available on request. The raw sequence data reported in this paper have been deposited in the Genome Sequence Archive (Genomics, Proteomics & Bioinformatics 2025) in National Genomics Data Center (Nucleic Acids Res 2025), China National Center for Bioinformation/Beijing Institute of Genomics, Chinese Academy of Sciences (PRJCA071042) that are publicly accessible at https://ngdc.cncb.ac.cn/gsa (accessed on 11 October 2026).

## Supplementary Materials

The following supporting information can be downloaded at https://www.mdpi.com/article/10.3390/biology15201835/s1, Figure S1. Effects of NH_3_ stress for 48 h on the histopathology of gill tissue in *Procambarus clarkii*. Figure S2. Melting curves of primers for candidate reference genes. Figure S3. Comparison of RNA-seq and qPCR expression changes for four NH_3_-responsive genes normalized using different reference genes. Figure S4. The distribution range of Ct values of candidate reference genes under four different conditions. Table S1. Expression stability analysis of 8 candidate and 5 traditional reference genes in the hepatopancreas transcriptome under NH_3_ stress. Table S2. Accession numbers and amplification efficiencies of the candidate reference genes used for qPCR analysis.
