# Supplementary material for: Systematic Screening of Reference Genes for qPCR in Procambarus clarkii: RPL13a Shows Stable Expression Under Non-Ionic Ammonia Stress and Multiple Experimental Conditions
Source: Biology (Basel). 2026 Oct 13;15(20):1835. doi: 10.3390/biology15201835 (PMC13701790; doi:10.3390/biology15201835)
Supplement: Supplementary file 1 [file biology-15-01835-s001.zip › biology-4587816-supplementary.pdf]

Figure Legends

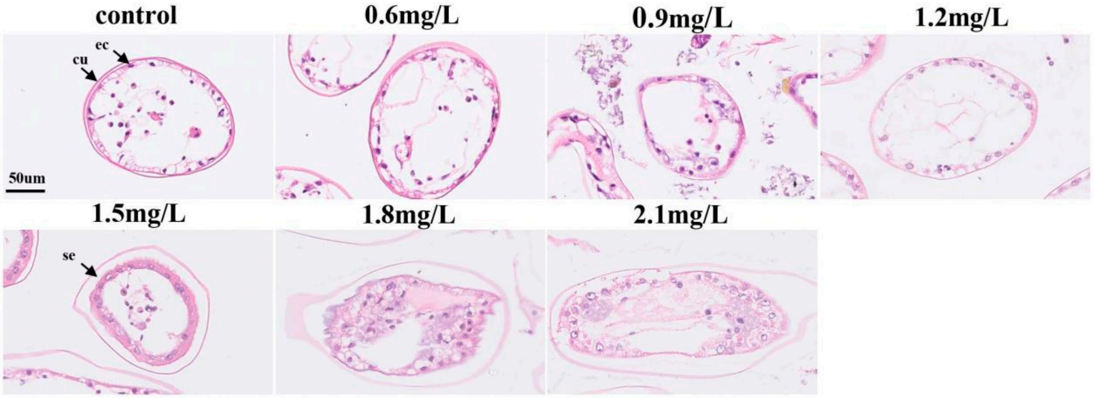

Figure S1. Effects of NH_3 stress for 48 h on the histopathology of gill tissue in *Procambarus clarkii*.

cu: cuticle; ec: gill epithelial cell; se, separation of respiratory epithelial cells from the cuticle.

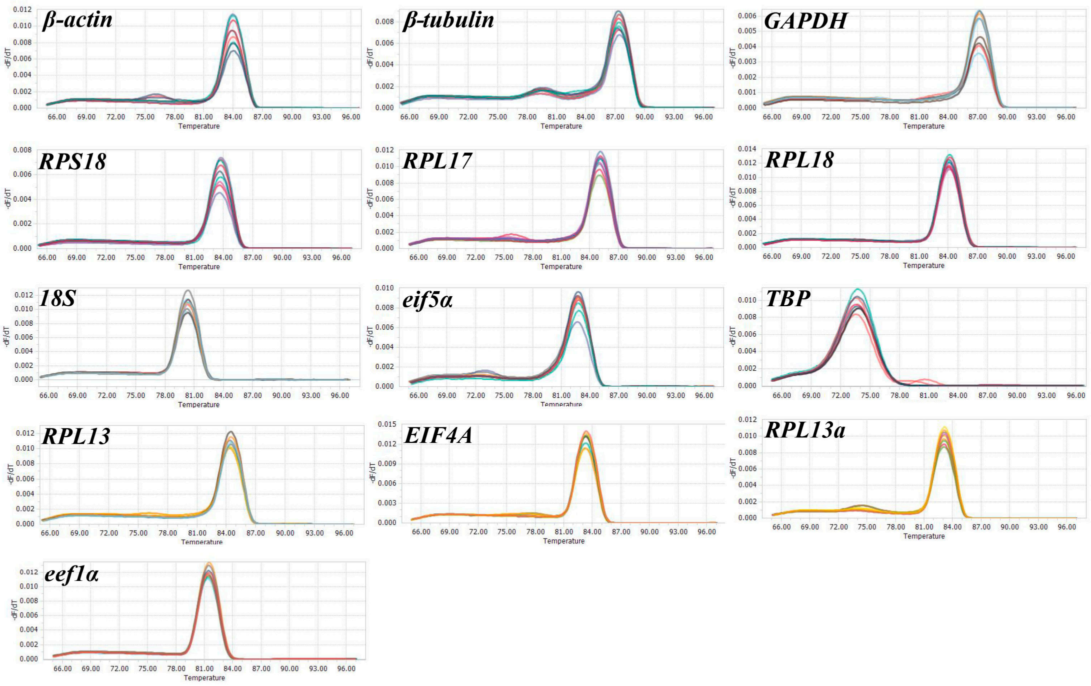

Figure S2. Melting curves of primers for candidate reference genes.

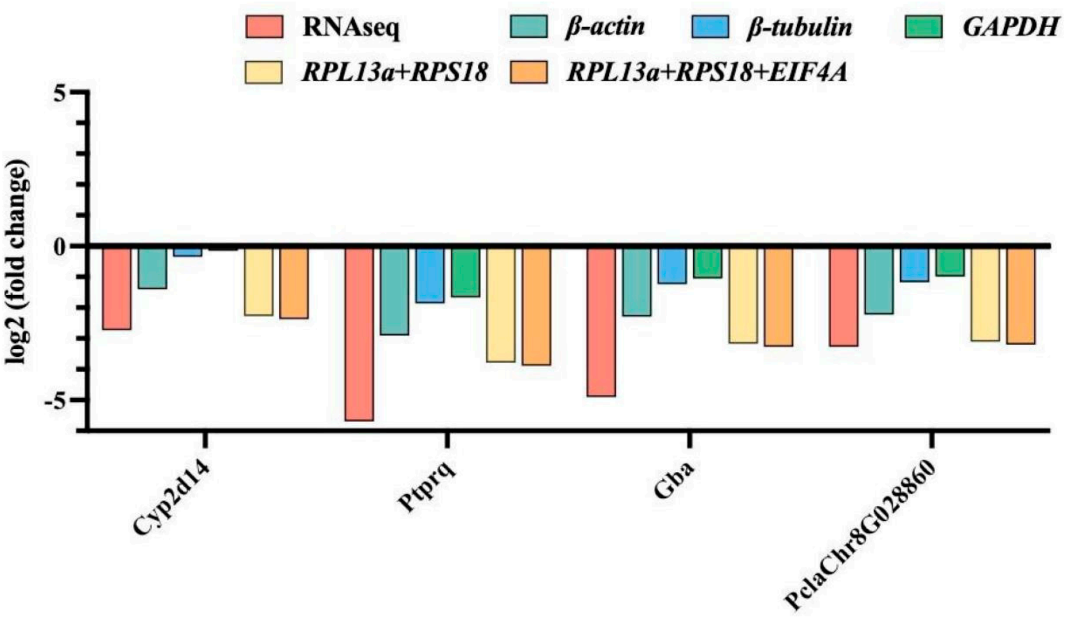

Figure S3. Comparison of RNA-seq and qPCR expression changes for four NH_3-responsive genes normalized using different reference genes.

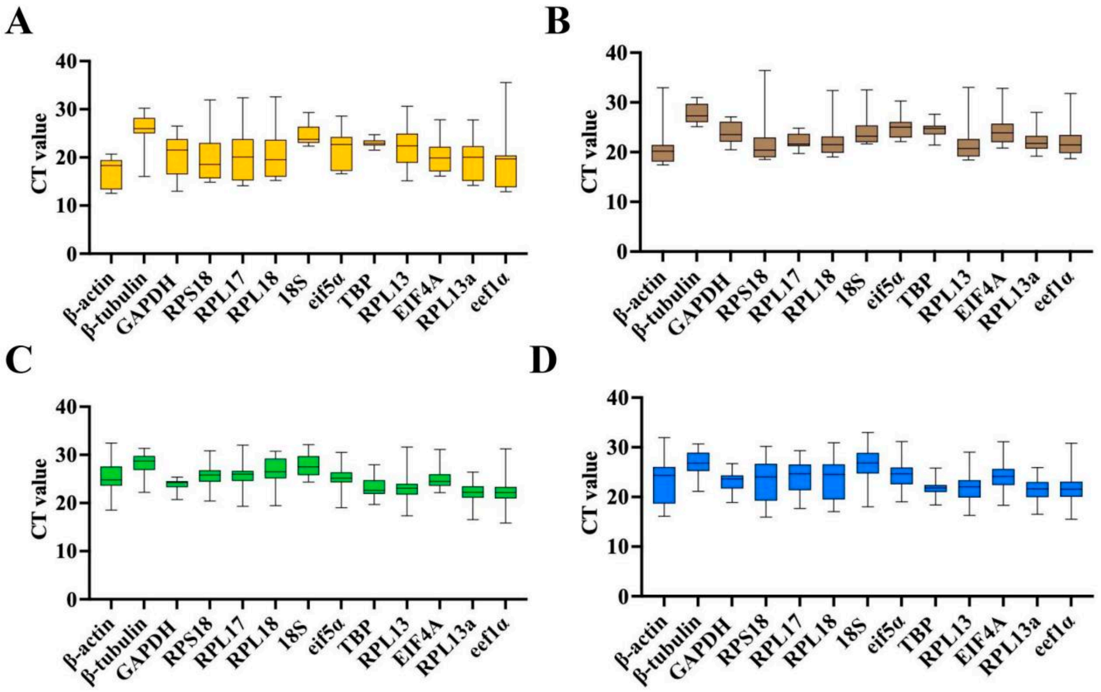

Figure S4. The distribution range of Ct values of candidate reference genes under four different conditions.

(A) Embryos at eight different developmental stages. **(B)** Ovaries at six different developmental stages. **(C)** Eight different tissues from both male and female individuals. **(D)** Eight different tissues following white spot syndrome virus (WSSV) infection.

Table S1. Expression stability analysis of 8 candidate and 5 traditional reference genes in the hepatopancreas transcriptome under NH_3 stress.

Gene name	Con-1	Con-2	Con-3	NH_3 tr-1	NH_3 tr-2	NH_3 tr-3	Mean	Stdev	CV value
β-actin	0.00	0.00	0.00	0.04	0.03	0.00	0.01	0.02	1.61
β-tubulin	0.00	0.00	0.00	2.16	1.81	1.60	0.93	1.03	1.11
GAPDH	9.54	9.64	9.95	9.62	9.55	9.53	9.64	0.16	0.02
RPS18	11.18	10.98	10.57	11.30	11.28	11.36	11.11	0.30	0.03
RPL17	9.79	10.21	9.71	10.09	10.16	10.26	10.04	0.23	0.02
RPL18	10.21	10.75	10.63	10.74	10.71	10.72	10.63	0.21	0.02
18S	0.00	0.00	0.00	4.87	4.70	4.86	2.41	2.64	1.10
eif5α	6.67	6.74	8.01	7.28	7.11	7.15	7.16	0.48	0.07
TBP	0.00	0.00	0.00	0.85	1.17	0.00	0.34	0.53	1.58
RPL13	10.75	10.62	11.16	11.19	11.20	11.18	11.02	0.26	0.02
EIF4A	8.56	8.70	8.57	9.13	9.03	8.92	8.82	0.24	0.03
RPL13a	10.92	10.71	11.00	11.09	11.07	11.06	10.97	0.14	0.01
eef1α	3.07	0.00	0.00	2.08	1.80	2.09	1.51	1.24	0.83

Mean Expression: Average Log2(FPKM+1) values from RNA-seq data.

SD: Standard Deviation of expression levels across all samples.

CV: Coefficient of Variation, calculated as SD/Mean

(SD/Mean)×100% . Lower CV indicates higher stability.

Con: Control group; NH3: Non-ionized ammonia treatment group.

Table S2. Accession numbers and amplification efficiencies of the candidate reference genes used for qPCR analysis.

Gene name	Accession number	Efficiency (%)
β-actin	KR135165.1	108.0
β-tubulin	KR135168	97.5
GAPDH	AB094145.1	94.5
RPS18	XM_045769132	104
RPL17	XM_045739168	96.0
RPL18	XM_045761550	98.0
18S	XM_045748331	97.5
eif5α	KR135170	95.0
TBP	KR135169	96.0
RPL13	XM_045738063	99.0
EIF4A	MK244964	94.5
RPL13a	XM_045729582	105.0
eef1α	MF385057	108.0
